# A New Software for Quantifying Motor Deficit After Stroke: A Case–Control Feasibility Pilot Study

**DOI:** 10.3389/fneur.2021.603619

**Published:** 2021-02-11

**Authors:** Raquel Gutiérrez Zúñiga, María Alonso de Leciñana, Alejandro Díez, Gabriel Torres Iglesias, Alejandro Pascual, Ariaki Higashi, Jorge Rodríguez Pardo, David Hernández Herrero, Blanca Fuentes, Exuperio Díez Tejedor

**Affiliations:** ^1^Department of Neurology and Stroke Center, Hospital La Paz Institute for Health Research-IdiPAZ, La Paz University Hospital, Universidad Autónoma de Madrid, Madrid, Spain; ^2^System Friend Inc., Hiroshima, Japan; ^3^Escuela Técnica Superior de Ingenieros de Telecomunicación, Universidad Politécnica de Madrid, Madrid, Spain; ^4^Department of Rehabilitation, Hospital La Paz Institute for Health Research-IdiPAZ, La Paz University Hospital, Universidad Autónoma de Madrid, Madrid, Spain

**Keywords:** motion capture system, kinematic metrics, stroke outcome, motor evaluation, rehabilitation

## Abstract

**Introduction:** The degree of disability after stroke needs to be objectively measured to implement adequate rehabilitation programs. Here, we evaluate the feasibility of a custom-built software to assess motor status after stroke.

**Methods:** This is a prospective, case–control pilot study comparing stroke patients with healthy volunteers. A workout evaluation that included trunk and upper limb movement was captured with Kinect® and kinematic metrics were extracted with Akira®. Trunk and joint angles were analyzed and compared between cases and controls. Patients were evaluated within the first week from stroke onset using the National Institutes of Health Stroke Scale (NIHSS), Fulg-Meyer Assessment (FMA), and modified Rankin Scale (mRS) scales; the relationship with kinematic measurements was explored.

**Results:** Thirty-seven patients and 33 controls were evaluated. Median (IQR) NIHSS of cases was 2 (0–4). The kinematic metrics that showed better discriminatory capacity were body sway during walking (less in cases than in controls, *p* = 0.01) and the drift in the forearm–trunk angle during shoulder abduction in supination (greater in cases than in controls, *p* = 0.01). The body sway during walking was moderately correlated with NIHSS score (Rho = −0.39; *p* = 0.01) but better correlated with mRS score (Rho = −0.52; *p* < 0.001) and was associated with the absence of disability (mRS 0–1) (OR = 0.64; *p* = 0.02). The drift in the forearm–trunk angle in supination was associated with the presence of disability (mRS >1) (OR = 1.27; *p* = 0.04).

**Conclusion:** We present a new software that detects even mild motor impairment in stroke patients underestimated by clinical scales but with an impact on patient functionality.

## Introduction

Stroke is the most prevalent cause of disability worldwide. Two of three stroke survivors will develop deficits that will cause high healthcare and social costs (1, 2). Apart from speech, visual, or cognitive deficits, one of the most important components of stroke-related disability is motor function impairment. Even mild deficits that may not be detected in routine clinical evaluation may significantly reduce patient's quality of life by interfering with the activities of daily living and their capacity to return to work. For these reasons, it is important to reliably measure these deficits and be able to correlate them with the degree of disability in order to implement adequate and personalized rehabilitation programs.

Motion capture systems (MCS) have been used to assess motor function in different neurological conditions with promising results (3–7). The main advantages are their low cost and relative ease of use. The most commonly used system is Microsoft Kinect®, which is a portable and marker-free motion capture system that uses an infrared light and a deep sensor to create a three-dimensional reconstruction of the human body and detect its movements. Kinect® results are concordant with marker-based systems which are the gold standard for motion analysis (8). In combination with specific software, Kinect® can be used for rehabilitation purposes (9). Previous studies have evaluated the feasibility of this system for gait assessment in multiple sclerosis or Parkinson disease (5, 7, 10) and for upper extremity motor function evaluation in muscle diseases (3, 4).

The use of kinematic metrics as a reliable measure of motor function for rehabilitation purposes in stroke patients is currently recommended (11). However, only few studies evaluate the usefulness of kinematic measurements to analyze motor deficit after stroke in order to help physicians to objectively measure patient's deficits. One example is the KINARM system that evaluates upper limb function in stroke patients (12, 13). Nevertheless, this system is complex and requires an exoskeleton, making it unsuitable to be used in routine clinical practice. Recent works suggest that Kinect® and virtual reality systems can effectively guide rehabilitation workouts in stroke patients (9, 14), but few studies analyze the usefulness of the Kinect® system for assessing poststroke functional status. One includes gait assessment (6), while others analyze reaching tasks in poststroke patients and show good concordance with specific clinical scales (15, 16). However, to our knowledge, a complete workout design to test the global function in poststroke patients with Kinect® has not been studied, and there is no information available about the potential relationship between the kinematic measures and disability after stroke.

Although Kinect® is becoming widely used, each research group uses their own software for the kinematic analysis. The Akira® software (Akira, System Friend Inc.) is a custom-built software developed to be used with Kinect® without body markers, which reconstructs a three-dimensional avatar of the human body and obtains kinematic metrics from body movement records.

Our main aim is to evaluate the usefulness of Microsoft Kinect® along with the software Akira® for an objective motor status evaluation after stroke. The secondary objective is to explore the relationship between kinematic metrics provided by the software and functional status after stroke.

## Materials and Methods

This is a case–control, pilot-feasibility study. Cases were prospectively selected from in-hospital patients with the diagnosis of acute ischemic stroke, able to stand up, and without significant language disorder that could interfere with the understanding of the purpose of the study and the explanation of the workout. Controls were subjects without any neurological condition or osteomuscular diseases. Controls were not paired with cases. The sample size was estimated for at least 30 participants in each group due to its exploratory nature.

**Figure 1:**
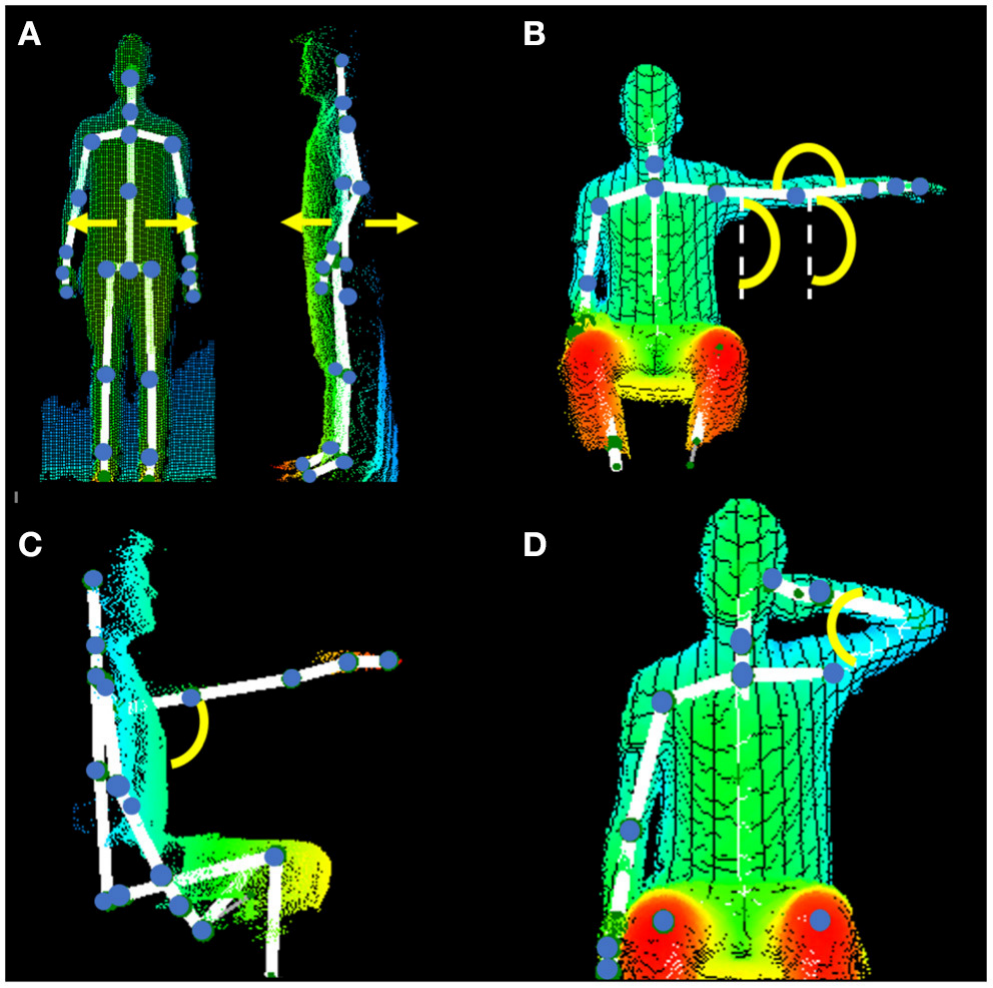
Three-dimensional representation of the body obtained with the Kinect–Akira system and angles of interest. **(A)** Exercises 1 to 4 in the frontal plane (left) and in the sagittal plane (right). We calculated the body oscillation (body sway) as the angle between the maximum trunk deviation to the left and to the right with respect to baseline position during the exercise. **(B)** Exercises 5 and 6 in the frontal plane. Extracted from the Fugl–Meyer Assessment (FMA). **(A)** Upper extremity: seating position. III. Volitional movement mixing synergies, shoulder flexion 0–90° and pronation supination: In this case, we asked the participant to perform an abduction movement from 0 to 90° and maintain the position during 5 s with the elbow in pronation. After 5 s, we asked the participant to change to elbow supination and maintain the position for another 5 s. Angles measured are shoulder with the trunk, elbow in extension, and forearm with the trunk. We measured the difference between the start and the end (after 5 s) of the arm position. **(C)** Exercise 7. Extracted from the FMA. **(A)** Upper extremity: seating position. IV. Volitional movement with little or no synergy: shoulder flexion 90–180°. We asked the participant to perform a shoulder flexion from 0 to 90° and from 90 to 180°. We measured the maximal angle of the shoulder with the trunk reached in the sagittal plane. **(D)** Exercise 8. Extracted from the FMA. II. Volitional movements within synergies. Flexor synergy: We asked the participant to touch the ipsilateral ear. We measured the minimum angle of the elbow flexion reached in the frontal plane.

We designed a workout extracted from the Fugl–Meyer Assessment (FMA) motor section (17) to be captured with Kinect® (Kinect 2 for Windows, SDK 2.0). The kinematic metrics were obtained with the Akira® software. The workout, kinematic metrics, and FMA item of reference are described in Table 1 and exemplified in Figure 1. Briefly, the workout comprises eight exercises that were performed always in the same order.

**Table 1:** Description of the exercises and calculation formula for each parameter.

**Exercise**	**Plane**	**Angles evaluated**	**Measurements**
1. Standing position 10 s	Frontal and sagittal	Axial trunk angle	ABS = MaxTA–MinTA
2. Walking 2 m	Frontal and sagittal	Axial trunk angle	ABS = MaxTA–MinTA
3. Sitting position, eyes open	Frontal and sagittal	Axial trunk angle	ABS = MaxTA–MinTA
4. Sitting position, eyes closed	Frontal and sagittal	Axial trunk angle	ABS = MaxTA–MinTA
5. Shoulder abduction with forearm in pronation (5 s) → arm drift^*^	Frontal	Shoulder angle, elbow angle, forearm–trunk angle	ΔSAp = SAps0–SAps5 ΔFAp = FAps0–FAps5 ΔEAp = EAps0–EAps5
6. Shoulder abduction with forearm in supination (5 s) → arm drift^*^	Frontal	Shoulder angle, elbow angle, forearm–trunk angle	ΔSAs = SAss0–SAss5 ΔFAs = FAss0–FAss5 ΔEAs = EAss0–EAss5
7. Shoulder flexion at 90° and 180°^**^	Sagittal	Shoulder angle	Maximum shoulder angle reached
8. Elbow flexion with shoulder in abduction^***^	Frontal	Elbow angle	Minimum elbow angle reached

*ABS, average body sway; MaxTA, maximum trunk angle; MinTA, minimun trunk angle; Δ, variation; SAp, shoulder angle in pronation; SAs, shoulder angle in supination; FAp, forearm angle in pronation; FAs, forearm angle in supination; EAp, elbow angle in pronation; EAs, elbow angle in supination; s0, second 0; s5, second 5. These measurements are graphically shown in Figure 1. The average body sway is defined as the body oscillation and calculated as the angle between the maximum trunk deviation to the left and to the right with respect to baseline position during the exercise*.

* *Extracted from the Fugl**–**Meyer Assessment (FMA). A. Upper extremity: seating position. III. Volitional movement mixing synergies, shoulder flexion 0–90° and pronation supination: In this case, we asked the participant to perform an abduction movement from 0 to 90° and maintain the position during 5 s with the elbow in pronation. After 5 s, we asked the participant to change to elbow supination and maintain the position for another 5 s*.

** *Extracted from the FMA. A. Upper extremity: seating position. IV. Volitional movement with little or no synergy: shoulder flexion 90–180°. We asked the participant to perform a shoulder flexion from 0 to 90° and from 90 to 180°*.

*** *Extracted from the FMA. II. Volitional movements within synergies. Flexor synergy: We asked the participant to touch the ipsilateral ear*.

The first four exercises were designed to evaluate the trunk balance: (1) standing for 10 s, (2) walking 2 m, (3) sitting position with eyes opened for 5 s, and (4) with eyes closed for 5 s. For these exercises, we calculated body oscillation (body sway) as the angle between the maximum trunk deviation to the left and to the right with respect to baseline position during the exercise. The other four exercises of the workout were designed to evaluate the upper limb movement. Participants were asked to perform a shoulder abduction reaching 90° with forearm in pronation and to maintain the position for 5 s. Then, they were asked to change the forearm to supination and to maintain the position for 5 s. For these two exercises, we calculate the drift in the shoulder–trunk angle, elbow–trunk angle, and forearm–trunk angle from the start to the end of each position. Then, we asked the participant to perform a shoulder flexion to 90 and 180°, and we measured the maximum shoulder angle reached. Finally, we asked the participant to reach the ear with the ipsilateral hand and calculated the minimum elbow angle reached. For arm exercises, we used the difference in performance between the affected side and the normal side in each subject in order to reduce individual variability; so for every angle, the absolute difference between both sides was used. The total workout duration estimated was 1 min and 30 s. Every exercise was explained to the participants before execution and they were video-guided during the recording. Also, the participants were guided by the clinician during the workout to ensure that it was correctly performed. Each participant performed the workout just once, in order to reduce the likelihood of practice effects.

Variables recorded were demographics and characteristics of stroke for cases. Stroke severity was assessed by certified neurologists and a rehabilitation physician using the National Institutes of Health Stroke Scale (NIHSS) and the FMA upper limb sections II, III, and IV (17), and functional status was measured using the modified Rankin Scale (mRS) score (18). All patients were clinically evaluated at the same time of kinematic evaluation. Informed consent was obtained from each participant. The study was approved by the Research ethics committee with Medical Products of La Paz University Hospital.

The statistical analysis was performed using SPSS 12.0 for Windows (SPSS Inc., Chicago, IL). The descriptive and comparative analyses were conducted considering the predefined groups. Categorical variables were expressed as percentages and compared between groups using the chi-square test. Continuous variables were expressed as mean and standard deviation (SD) or median and interquartile ranges (IQR) and were compared using Student's *t*-test.

Univariate and multivariate regression analyses adjusted by age and sex were performed to identify those kinematic measurements that were independently associated with the diagnosis of stroke (cases). The variables included in the model were those that achieved a difference with *p* < 0.10 in the mean comparison tests.

Within the cases group, a Spearman correlation analysis was performed to explore the association between kinematic metrics that were independently associated with the diagnosis of stroke and the clinical scales scores at the time of evaluation. Additionally, the association of kinematic metrics and the presence of disability (defined as mRS score 2–5 vs. no disability: mRS 0–1) was analyzed using a logistic regression analysis.

All tests were two-tailed, and statistical significance was established as *p* < 0.05.

The data that support the findings of this study are available from the author for correspondence upon reasonable request.

## Results

From March 2017 to November 2017, 70 participants were enrolled. A total of 37 ischemic stroke cases and 33 controls were included. Cases were significantly older than controls (mean ± SD: 70.4 ± 11.216 vs. 55.5 ± 12.3 years; *p* < 0.001) and were more frequently men (64.8 vs. 27.3%; *p* = 0.002). Cases had mild strokes with median NIHSS 2 (IQR 0–4), median motor NIHSS subscale 1 (IQR 0–2), and median FMA 18 (IQR 13–31). They also showed moderate disability with median mRS 2 (IQR 0–3) at the moment of the evaluation. The time lapse from stroke onset to the study evaluation was 3 (2–6) days [median (IQR)].

Comparison of the kinematic data between cases and controls is described in Table 2. Cases showed less body sway than controls in the frontal and sagittal planes when walking with almost half of the body oscillation grades and greater body sway in the sagittal plane while in sitting position. Cases showed greater arm drift than controls during the shoulder abduction, mainly in supination. After adjusting for age and sex, the drift in the forearm–trunk angle in supination (*B* coefficient 0.28; *p* = 0.04) and the reduced body sway in the frontal plane during walking (*B* coefficient −0.36; *p* = 0.01) remained significantly associated with stroke cases.

**Table 2:** Comparison of kinematic metrics between cases and controls.

**Measurements**		**Controls Mean degrees (SD) *N* = 33**	**Cases Mean degrees (SD) *N* = 37**	***p***
1. Standing position 10 s	Body sway. Frontal plane.	1.09 (0.86)	1.70 (2.27)	0.13
	Body sway. Sagittal plane.	3.12 (2.32)	2.49 (2.58)	0.29
2. Walking 2 m	Body sway. Frontal plane.	4.07 (2.73)	2.58 (2.06)	0.01
	Body sway. Sagittal plane.	4.57 (4.21)	2.61 (3.13)	0.02
3. Sitting position, eyes open	Body sway. Frontal plane.	1.62 (1.85)	1.85 (2.17)	0.64
	Body sway. Sagittal plane.	4.24 (4.24)	6.72 (5.11)	0.03
4. Sitting position, eyes closed	Body sway. Frontal plane.	1.64 (1.85)	2.12 (2.13)	0.33
	Body sway. Sagittal plane.	40.02 (56)	38.99 (50.5)	0.93
5. Shoulder abduction with forearm in pronation (5 s) → arm drift	Shoulder angle. Pronation. Frontal plane.	5.72 (15.14)	9.58 (12.15)	0.24
	Elbow extension angle. Pronation. Frontal plane.	2.60 (2.6)	11.52 (27.06)	0.05
	Forearm–trunk angle. Pronation. Frontal plane.	5.30 (14.7)	7.22 (9.3)	0.5
6. Shoulder abduction with forearm in supination (5 s) → arm drift	Shoulder angle. Supination. Frontal plane.	2.11 (1.99)	8.11 (17.93)	0.05
	Elbow extension angle. Supination. Frontal plane.	2.06 (2.66)	9.43 (25.94)	0.08
	Forearm–trunk angle. Supination. Frontal plane.	2.18 (1.86)	4.97 (4.2)	0.01
7. Shoulder flexion at 90° and 180°	Shoulder angle. Sagittal plane.	29.45 (45.9)	31.83 (33.6)	0.8
8. Elbow flexion with shoulder in abduction	Elbow flexion angle. Frontal plane.	17.63 (32.6)	16.74 (19.9)	0.89

*Data for exercises exploring arm movements show the difference in the measurements of angles (degrees) between both sides*.

Body sway in the frontal plane during walking was moderately correlated with the NIHSS score (Rho = −0.39; *p* = 0.01) but more strongly correlated with the mRS (Rho = −0.52; *p* < 0.001). The drift in forearm–trunk angle was not correlated with any of the scales (correlations are shown in Table 3). We did not find any correlation between FMA score and kinematic data.

**Table 3:** Spearman's Rho correlation between National Institutes of Health Stroke Scale (NIHSS), motor NIHSS subscale, Fugl–Meyer Assessment (FMA), and modified Rankin Scale (mRS) and the drift in forearm–trunk angle in supination and body sways during walking.

	**Body sway in frontal plane**		**Drift in forearm–trunk angle**	
	**Rho**	***p***	**Rho**	***p***
NIHSS	−0.39	0.01	0.09	0.57
NIHSS motor subscale	−0.31	0.06	0.05	0.76
mRS	−0.52	<0.001	0.29	0.07
FMA	0.06	0.72	0.06	0.73

In the logistic regression analysis, we found that the drift in the forearm–trunk angle in supination was associated with the presence of disability (OR = 1.27; 95% CI = 1.01–1.60, *p* = 0.04) and that body sway during walking was associated with the absence of disability (OR = 0.64; 95% CI = 0.43–0.93; *p* = 0.02).

## Discussion

The results of this pilot study suggest that this novel custom-built software allows discrimination of mild deficits in stroke patients in comparison with subjects without stroke, even though these findings show a poor correlation with the score in the NIHSS or the FMA. This suggests that the tool can detect minor deficits not quantified by the clinical scale but which can impact patient's functionality as suggested by the better correlation between the kinematic metrics and the mRS score.

Kinect® has previously been proven useful in the assessment of other neurological conditions such as posture disorders associated with multiple sclerosis, gait disorders in Parkinson's disease or the upper limb movement in type III Spinal muscular atrophy or amyotrophic lateral sclerosis (3–6). Kinect® is also widely used in stroke rehabilitation, together with video games for therapy purpose (9, 19). Apart from the easy use of Kinect®, its main advantages when used with the software Akira® is that the workout chosen to evaluate motor function can be personalized for specific needs. Moreover, it is portable and cheap, thus being more accessible and suitable for clinical evaluation at the patient bedside. All these characteristics make this tool suitable to be used in the neurological clinical setting for the objective evaluation of motor function in acute stroke patients if the promising results of this pilot study are confirmed and better established in further studies.

The measures that showed the greatest differences between cases and controls were reduction in body sway during walking and forearm–trunk angle drift during shoulder abduction in supination. The latest might be expected due to motor impairment after stroke, but it is remarkable that most of the cases included in this pilot study had very mild deficits that may not be quantified during the routine clinical evaluation. These results are in agreement with other studies of stroke patients, in which subclinical motor deficits were found using robotic technology such as KINARM (12, 13).

The reduction in body sway found among stroke patients is a novel and interesting finding, since it is not routinely assessed. Musculoskeletal disorders, age, and other non-neurological conditions may affect gait. Different walking patterns have been described in subjects without neurological disorders as well as in Parkinson's disease and in stroke using Kinect® (using signal processing and measurements different from those used in this study) (6). Our results suggest that walking patterns can be altered after a stroke even in patients without a significant motor deficit.

Moreover, the body sway during walking had a weak correlation with the NIHSS score and a strong correlation with the mRS: the less body sway, the more disability. Interestingly, the reduction in body sway during walking and the drift of forearm–trunk angle during shoulder abduction in supination were also associated with the presence of disability. This is the first time, to our knowledge, that specific kinematic metrics show association with the functional status of the patient after stroke and, therefore, deserve further investigation.

Apart from the low sensitivity of the neurological scales routinely used in clinical practice to accurately measure minor motor deficits, another disadvantage of these scales is that the scoring relies on the subjective assessment of the evaluator, which may reduce the accuracy of the assessment especially for minor deficits. Also, the various motor scales are strongly correlated between them, but weakly correlated with disability scales (20), and thus, certain motor deficits which have a real impact on the quality of life of the patient could be underestimated. All these disadvantages of clinical scales could be overcome using a reliable motion capture system capable of being used at the bedside. The poor correlation of the kinematic metrics with the clinical scales for quantification of the neurological deficits (NIHSS, FMA) and the better correlation with disability assessed by the mRS in this pilot study suggest that this could be a good tool for clinicians to evaluate subtle deficits that have an impact on the patient's functional status. This could be important in clinical practice, as the prescription of an occupational therapy or rehabilitation program is usually made based on clinical evaluation.

Our study has some limitations. The most important is the large difference in age and gender between the two study groups. Although these variables were considered in the multivariate analysis, future studies should correct this methodological limitation. Also, the small sample size prevented adjustment by other confounders such as stroke location, which may influence results. On the basis of the data obtained from this pilot study, a larger one will be performed to confirm the findings and to determine the clinical applicability of this tool for the assessment of motor impairment and disability in stroke patients in clinical practice. In this regard, we have designed the AKIRA II study (ClinicalTrials.gov identifier: NCT04464863) with a larger sample size and a prospective design that will match cases and controls by age and sex and will analyze results according to stroke location and characteristics in brain MRI.

In conclusion, we present a new software that detects motor impairment even in patients with mild strokes and motor deficits that are underestimated by clinical scales but have an impact on patient functionality. Given the fact that stroke is one of the main causes of disability, this tool could help the physician to globally evaluate patient's motor function easily at the bedside and, eventually, recommend rehabilitation therapy based on this objective evaluation.

These promising results should be validated with further studies to better establish their clinical applicability, with the inclusion of more kinematic metrics (as movement acceleration) and the creation of population-based normalized kinematic data per age range, based on current recommendations (11, 21).

## Data Availability Statement

The raw data supporting the conclusions of this article will be made available by the authors, without undue reservation.

## Ethics Statement

The studies involving human participants were reviewed and approved by the Research Ethics Committee with Medical Products of La Paz University Hospital. The patients/participants provided their written informed consent to participate in this study.

## Author Contributions

RG: acquisition of data, study concept, analysis and interpretation, and preparation of the manuscript. MA: acquisition of data, study concept, analysis and interpretation, and critical revision of the manuscript. DH: design of the workout evaluation and critical revision of the manuscript. AP: design of the workout evaluation, technical support, analysis of data, and critical review of the manuscript. AD and AH: design and technical support of the Akira software. GT and JR: acquisition of data and critical revision of the manuscript. BF: study concept, analysis and interpretation, and preparation of the manuscript. ED: study concept, analysis and interpretation, and critical revision of the manuscript. All authors contributed to the article and approved the submitted version.

## Conflict of Interest

AD and AH were employed by the company System Friend Inc. The remaining authors declare that the research was conducted in the absence of any commercial or financial relationships that could be construed as a potential conflict of interest.
